# Nurses’ perspectives on caregiver engagement in hematopoietic stem cell transplantation: A qualitative study

**DOI:** 10.1007/s00520-026-10424-4

**Published:** 2026-02-11

**Authors:** Nese Altınok Ersoy, Esra Atakul, Imatullah Akyar

**Affiliations:** 1 Hacettepe University Faculty of Nursing, Adnan Saygun Str., Block D, 2nd Floor, Office No: 33-29 Sihhiye, Ankara, 06230 Turkey; 2 Saglık Bilimleri University Dr. Abdurrahman Yurtaslan Ankara Oncology Training and Research Hospital, Ankara, Turkey

**Keywords:** Caregivers, Hematopoietic stem cell transplantation, Nurses, Qualitative research

## Abstract

**Purpose:**

Hematopoietic stem cell transplantation (HSCT) is a physically and psychosocially complex process for patients and their families, and nurses play a key role in coordinating patient care. This study aimed to explore nurses’ perspectives on caregiver engagement in the care of patients undergoing hematopoietic stem cell transplantation.

**Methods:**

A qualitative descriptive design with purposive sampling was used. Data were collected through semi-structured interviews with eleven nurses working in a bone marrow transplantation unit in Ankara. The data were analyzed using thematic analysis.

**Results:**

Five main themes and ten sub-themes were identified. The main themes were 1) Nurses’ expectations and caregiver involvement, 2) Impact of caregiver’s engagement, 3) Caregiver strain and challenges, 4) Support needs of caregivers, and 5) Nurses’ role in supporting caregivers.

**Conclusions:**

Nurses perceived that caregiver engagement positively improves patient outcomes but also noted that this placed a significant burden on caregivers. Nurses play a pivotal role in addressing the challenges and supporting caregivers through emotional support, education, and advocacy. Targeted interventions, such as education, psychological support, and enhanced communication, are crucial for supporting caregivers effectively and improving the well-being of both caregivers and patients.

**Supplementary information:**

The online version contains supplementary material available at 10.1007/s00520-026-10424-4.

## Introduction

Hematopoietic stem cell transplantation is a highly prevalent, complex, and demanding process, characterized by treatment-related toxicity, life-threatening complications, and extended hospital stays [1–3]. As of March 2025, 889,500 patients were registered in the European Society for Blood and Marrow Transplantation-EMBT database, with 43,902 transplants reported in 2023. Despite its therapeutic promise, this process is characterized by treatment-related toxicity, life-threatening complications, and prolonged hospitalizations [4]. Patients undergoing HSCT often experience profound physical, emotional, and psychological distress due to the aggressive nature of the treatment and the prolonged recovery period [5]. Caregivers play a vital role not only in managing physical aspects of care, such as medication administration and symptom monitoring, but also in providing essential emotional and psychological support during a highly stressful and uncertain time [4]. With this challenging context, nurses and caregivers work collaboratively to deliver comprehensive care [6]. Also, nurses play a unique and central role as the “bridge between the institution and the family” [7, 8].

The existing literature comprehensively addresses the critical role played by caregivers and the significant challenges they face [9]. Studies highlight the diverse burdens and responsibilities experienced by caregivers, including social isolation, emotional exhaustion, changes in family dynamics, and infection control fear of recurrence, anxiety, and depression [9, 10]. Furthermore, studies [11–13] emphasize that supporting caregivers is essential for optimal patient care quality. From pre-transplant preparation through post-transplant recovery, effective communication and collaboration among caregivers, nurses, and the healthcare team are crucial to ensure optimal patient outcomes [7, 8]. Early studies, such as one from 1996, also underscored the importance of active caregiver participation in care planning and implementation [14] as well as effective communication with the healthcare team [7, 8].

However, the success of the process depends mainly on the caregiver’s ability to assume complex responsibilities and manage the associated burden [15–17]. Nurses are the ones who provide the necessary education to caregivers, manage their concerns, and facilitate their adaptation to the process. [6]. Therefore, the perceptions and experiences of nurses, who are central to and manage this process, regarding caregiver engagement are vital for understanding the quality of care. While existing studies [15–18] have focused on the experiences or needs of caregivers, a significant gap remains in the literature regarding studies that examine caregiver engagement from the perspective of nurses and their lived experiences [19, 20]. Insights are essential for developing and revisiting holistic care models that recognize and empower caregivers, while also enhancing patient and caregiver outcomes throughout the transplant journey [21, 22]. Therefore, this study aims to explore nurses’ experiences with caregiver engagement in patients undergoing hematopoietic stem cell transplants.

## Methods

### Study design

The study employed a qualitative descriptive design using semi-structured interviews to explore nurses’ perspectives of caregiver engagement in hematopoietic stem cell transplantation care. The qualitative descriptive design is the appropriate methodology for understanding subjective experiences from the participants’ perspectives [23]. The research question necessitated this approach because the study aimed to directly address the "what," "how," and "where" of nurses’ perspectives. This methodology, which focuses on direct description, was selected over interpretative methods such as phenomenology (which seeks the "lived essence" of experience) or grounded theory (which aims to generate abstract theories) [24, 25].

This study aimed to answer the following research question:

- How do nurses perceive the engagement of caregivers in the care of patients undergoing HSCT?

### Participants and setting

The study was conducted in a large academic tertiary center with a dedicated HSCT unit that performs approximately 90 HSCT (allogeneic and autologous) transplants annually. The study employed a purposive sampling method, which enabled the selection of participants capable of providing rich and in-depth information related to the research question. The sample consisted of nurses employed in the HSCT unit, who had at least one year of clinical experience. Eleven nurses participated in the study voluntarily. The sample size was determined by considering standard practices in similar qualitative studies [26] and the richness of the data. A scheduled meeting was conducted with the nurses, facilitated by the responsible nurse, to introduce and explain the research before the interviews. After all interviews were completed, the thematic analysis revealed that themes and codes were repeated among the participants, indicating data saturation had been achieved.

Inclusion criteria for participation in the study were as follows:

- Clinical experience in an HSCT unit for a minimum of one year.
- Direct clinical involvement in patient and caregiver care.
- Provision of informed consent and voluntary agreement to be interviewed.

### Data collection

Data were collected from January to March 2019. After agreeing to participate, participants completed a five-question socio-demographic form (age, experience, etc.), which was collected solely to define the sample and not to address the main research question.

The semi-structured interviews were conducted in a private room in the department. Semi-structured interviews were used because they ensured that all participants were asked the same basic questions using a pre-prepared guide, and probing questions at key points allowed for deeper understanding. Two trained female researchers (R1 & R2) conducted interviews. R1, a nurse academic with clinical experience in hematological malignancies and qualitative research, served as the primary interviewer. R2, with qualitative research experience in a non-clinical setting, monitored the interview process, took field notes, and recorded the interview [24, 25].

Each interview lasted about 20–25 min. Field notes were used as supplementary data and for early reflection. The second researcher took descriptive field notes (e.g., body language, setting) during each interview, and these notes were used in conjunction with the transcripts during analysis to enhance their depth. At the conclusion of each interview, researchers provided a verbal summary of key points to the participant to confirm whether the researcher had correctly understood the participant, as the data was still “fresh.” All participants confirmed that the summary accurately reflected their views.

A semi-structured interview guide (Box 1) was used. When developing the interview guide, a draft consisting of a pool of questions was first created based on a relevant literature review. The draft guide was presented to five experts (nursing experts with experience in caring for BMT patients) for review to improve the clarity and flow of the questions. Interviews and transcripts were conducted in Turkish. To preserve the cultural nuance and depth of meaning, the entire analysis (coding and thematization) was performed in Turkish. Only the final themes and representative quotes to be used in the article were translated into English. The guide provided consistency across interviews while allowing flexibility to explore emergent topics. Each researcher had a different personal perspective that influenced the analytical process. These diverse perspectives allowed us to interpret the data from multiple perspectives rather than from a single, narrow perspective, reducing bias, and themes were generated through team discussions (consensus).

**Box 1 **Interview Questions 

**Table:** 

• What do you think should be the roles of the caregivers during hospitalization? • Would you please discuss the caregivers’ participation in the care activities of patients? • How do you think the caregivers have an impact on the patient? Any positive or adverse effects? • To what extent do you expect the caregivers to participate in the patient’s care? When you think about your current patients and caregivers’, can you compare the real situation and your expectation? • Do you or other caregivers experience any problems during this time? What type of challenges do you face? • What do you think about the caregiver’s challenges in that period? • What do you think about the effect positive outcomes of caregiving in that period? • What are the support needs of caregivers? • As a nurse, what are you doing / can do for the support needs of caregivers?	

### Rigour

It is essential to clarify rigour to ensure that the process is trustworthy in qualitative design research. To achieve rigour in the data in this study, four criteria described by Guba and Lincoln (1989) were used.

Credibility: To ensure that the findings accurately reflect the participants’ experiences, member checking was conducted through a verbal summary provided by the researcher at the end of each interview. Furthermore, the inclusion of a third researcher with a **clinical experience** perspective in the analysis process enabled investigator triangulation and reduced subjectivity. The findings were enriched by supporting them with direct quotes from the participants [25].

Transferability: The study’s findings are not intended for generalization; instead, a rich and detailed description is provided to enable the reader to assess the validity of the findings within their own context. In this context, the participants’ demographic and professional characteristics (See Table 1) and the recruitment process are reported in detail.

**Table 1:** Socio-Demographic Characteristics of Participants

Participant	Age	Clinical Experience	HSCT Experience	Nursing Degree	Caregiving Experience	Marital Status
N1	23	6 years	3 years	BSN	No	Single
N2	30	8 years	5 years	MSN	Yes	Married
N3	35	8 years	8 years	BSN	Yes	Married
N4	30	12 years	10 years	BSN	No	Married
N5	28	6,5 years	4 years	BSN	Yes	Single
N6	40	20 years	10 years	High School	No	Married
N7	30	10 years	2 years	BSN	Yes	Married
N8	34	12 years	8 years	BSN	No	Married
N9	30	9 years	5 years	High School	Yes	Single
N10	33	8 years	4,5 years	BSN	Yes	Married
N11	30	10 years	4 years	‘BSN	Yes	Married

*BSN* Bachelor’s Degree *MSN* Master’s Degree

Confirmability: All researchers actively participated in the data analysis process independently.

Reliability: This was achieved by using a semi-structured interview guide based on literature and expert opinion to ensure consistency (Box 1) [24, 25]. The study adhered to the Standards for Reporting Qualitative Research (SRQR) guidelines (Supplementary File 1).

### Data analysis

Data were analyzed using the thematic analysis method described by Braun and Clarke [2006]. This approach was chosen because it aligns with the nature of a qualitative descriptive design [25]. All interviews were transcribed verbatim, and all transcripts were anonymized before analysis to ensure confidentiality. All interview recordings and transcriptions were stored on the encrypted computers of R1 and R2. MAXQDA 2020 was used in the data management process. The analysis process employed an inductive approach, with themes derived directly from the data. Researchers (R1, R2, R3) read and reread the transcripts and field notes to become familiar with the data. To ensure analytical depth, field notes and interview transcripts were triangulated to provide context to participants’ statements [24, 25]. The researchers first independently coded the transcripts and then met in regular analysis meetings. Differences in coding or interpretation were resolved through discussion and consensus.

To ensure reflexivity, non-leading, open-ended questions were asked to minimize interviewer bias, and assumptions were recorded in field notes [24].

The research team’s diverse professional positions (e.g., a senior clinical nurse and a qualitative methodologist) enriched the analysis. These diverse perspectives balanced biases in the analytical process and enabled a multidimensional interpretation of the data [24, 25].

### Ethical considerations

The study was approved by the Hacettepe University’s IRB (#18/770). The study was conducted in accordance with the Declaration of Helsinki. All participants were informed of the study purpose, their rights (including the right to withdraw at any time), and measures to ensure confidentiality. Participants agreed to be interviewed and signed a written informed consent form. The audio recordings, field notes, and software analysis protocols were stored in encrypted form on the first author’s computer. Participant identities were anonymized using unique codes (N1-N11).

## Results

### Participant characteristics

Eleven nurses participated in the study, with a mean age of 31.1 ± 4.33 years (range, 23–40). Most nurses held a bachelor’s degree (81.8%), with clinical experience ranging from 6 to 20 years (mean: 9.95 ± 3.86 years) and HSCT nursing experience ranging from 1 to 10 years (mean: 5.6 ± 3.05 years). More than half of the nurses (63.6%) reported having cared for a close family member (mother, father, mother-in-law, or father-in-law), and 56.5% were married.

### Themes and sub-themes

**Fig. 1:**
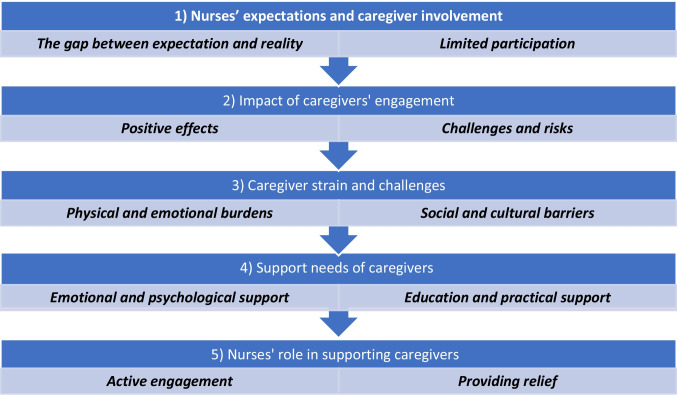
Caregiver engagement in HSCT care theme and sub-themes

Five main themes and ten sub-themes were identified (Fig. 1).

The main themes were 1) Nurses’ expectations and caregiver involvement, 2) Impact of caregivers engagement, 3) Caregiver strain and challenges, 4) Support needs of caregivers, and 5) Nurses’ role in supporting caregivers (Fig. 1).

### Nurses’ expectations and caregiver involvement

#### The gap between expectation and reality

Nurses expressed expectations that caregivers would actively engage in care, particularly in assisting with self-care and daily living activities. However, in practice, many noted a discrepancy between these expectations and the actual caregiver involvement. Nurses reported that:

*"I expect full support, but that seems impossible (N3)."*

*"They are not involved in the care, but that is my expectation (N4)."*

*"I do not expect them to be involved in the care and they are not interested in it. Caregivers help the patient with self-care (N6)."*

*"They should be involved in the primary care of the patient... (N7)."*

#### Limited participation

Nurses noted that, despite high expectations for engaging caregivers in patients’ daily activities, caregivers often provided minimal support. Nurses reported that:

*"... we expect them to follow the rules of the clinic,... while caring for the patient, it is important to take responsibility, we want them to follow the patient’s condition and inform us of any timely changes (N1)."*

*"They are not able to help with activities of daily living; they cannot meet their self-care needs, they have spent a month caring in a 4–5 square meter room... all of this has a negative impact on careers (N7)."*

### Impact of caregivers’ engagement

#### Positive effects

Nurses perceived that caregiver participation contributed to better hygiene, reduced hospital stays, and improved psychological well-being. Nurses reported that:

*"When caregivers are involved in care, discharge time is reduced, symptoms are better managed, and the patient receives better psychological support (N2)."*

*"The collaboration with the nurses always helps the patient and us; the course of the hospital stay is eased, the risk of infection is reduced, and the transplantation is accelerated (N4)."*

*"They learn the principles of caring for the patient and hygiene behavior (N3)."*

#### Challenges and risks

Nurses noted that caregivers sometimes overstep roles, attempt to interfere with medical decisions or procedures within the nurses’ purview, assume control, or become emotionally overwhelmed. Nurses reported that:

*“Caregivers need to pay attention to hygiene, they act uncontrolled, especially after allogeneic stem cell transplantation, they still need to pay attention to hygiene… However, the caregiver ignores hygiene. All rooms were cleaned carefully, but we stepped into some rooms one hour later, and we were surprised by what had happened (N6).”*

*"... caregivers start to see themselves as the main decision-makers in patient care, and they intervene in the care (N2)."*

*"Sometimes caregivers reflect the psychological state of the patients... they need help... working with the environmental organization, feeding the patients, caring for the patients can be good... (N2)."*

### Caregiver strain and challenges

#### Physical and emotional burdens

Nurses noted that when caregivers’ physical and emotional burdens increase, some consequences are reflected in patient care. They stated that nurses are unable to provide adequate emotional and care support to patients in these situations. Nurses reported that:

*"Caregivers support their patient psychologically, physically, and spiritually, but they feel exhausted and burdened... they accompany for a long time, and after a while they feel the longing for the child and the family and become unwilling to take care of the patient (N7)."*

*"Everyone expects everything from the caregivers... they have many needs, face a financial burden, family conflicts interfere with care, they came here from another city, we try to talk to them (N10)."*

*"They are stressed and nervous about the illness... we try to think positively, relax... I usually get results... I try to relax... psychological support is important (N8)."*

*“He does not want to take care of his patient because the treatment here is not just two days, but three days; it is sometimes a month, two months, or even three months. Today, one of the caregivers showed up and told us about the urgent need for a gynecology visit and treatment (N1).”*

*“When we visit the room, we can see that they are not eager to hand a glass of water… we do not want them to get out of the clinic, but they do (N4).”*

*“They are in a difficult situation... they have difficulty with managing the process, psychological effects. In one of the in-service trainings, our palliative nurse reminded the situation of caregivers... “Patients score pain as 5–6, whereas caregiver scores patients’ pain as 9–10... caregivers suffer more, they feel patients’ pain. With 9–10, they tell their suffering for the patient, score their distress, pain”... I think being a caregiver is tough (N6)”*

#### Social and cultural barriers

Nurses emphasized that family conflicts, cultural misunderstandings, and language barriers inhibit effective caregiving. Nurses reported that:

*"There are things that he (caregiver) considers right/correct in his (caregiver’s) culture; he has many wrong facts. They (caregivers) do not prefer to work together with their wrong beliefs and values (N2)."*

*"With some patients, we do not speak the same language, and as translators are not always available, we cannot communicate (N1)."*

*"They pretend they do not have a family or another life outside; they only focus on caring here (N5)."*

### Support needs of caregivers

#### Psychosocial and educational support

Nurses emphasized the importance of empathy, humor, and communication to ease caregiver stress. Nurses reported that:

*“… empathy... I think it is the most important thing… (N8)”*

*“communication and empathy. Telling that things will be over, saying hi/have a nice day… feels very nice... we should be in their shoes and act accordingly (N9)”*

*“We are chatting with them, we invite them, talk about how the things are going (N9)”*

*"We try to smile... we use humor. We try to make them forget that they are in hospital... we try to be warm and provide psychological support (N4)."*

*"We should be aware that the patients are on the shoulders of the caregivers and that caring for them means caring for the patients (N11)."*

*"Communication and empathy. Saying it will be over, saying hello/having a nice day... feels very good. We should put ourselves in their shoes and act accordingly (N9)."*

#### Education and practical support

Nurses stated that caregivers require training and support in areas such as hygiene, nutrition, and medication adherence. Nurses reported that:

*"Education of patients and caregivers is important... especially education about medication and nutrition is needed (N5)."*

*"We organize the environment for them and help them with bathing. We have routine psychologist visits for the patients; these visits also extend to the caregivers (N7)."*

*"We have teaching sessions, training sessions. Before hospitalization, colleagues in the outpatient department train caregivers and patients; they also receive feedback on the training. But here we also continue teaching, we keep telling them, nutrition, exercise, neutropenia (N1)."*

### Nurses’ role in supporting caregivers

#### Active engagement

Nurses described themselves as collaborators who observe, support, and listen to caregivers. *Nurses reported that:*

*"We all try to keep in touch with them, ask them questions. Inform them about occupational therapy activities. Our focus is on the patients; we encourage caregivers to share their experiences and discuss their problems. Observation is important; if we observe hopelessness, we talk to them, inform them about the process, and find solutions together (N11)."*

*"We try to be welcoming, warm, and communicate gently (N5)."*

*"We try to talk to them, we invite them, talk about how things are going (N9)."*

#### Providing relief

Nurses also stated that they provide breaks and relief for caregivers with under strain.* Nurses reported that:*

*"When we see the strain, we give them a break, help them to relieve themselves, we do not force them to do anything (N3)."*

*"We organize social activities for the caregivers and try to take their minds off caregiving for a while... we try to offer them a chance to relax and cope (N10)."*

## Discussion

This study aims to provide an understanding of nurses’ perceptions regarding caregiver engagement in HSCT care. The findings highlight both the critical role caregivers play in supporting patients during hospitalization and the challenges nurses observe in facilitating effective caregiver involvement. This study offers insights into nurses’ perceptions of caregiver engagement, the challenges caregivers face, and the support they require throughout the HSCT process.

A key focus of this study was the expectations nurses hold regarding caregiver participation, especially in tasks such as hygiene assistance, emotional support, and adherence to infection control measures. The findings align with previous literature highlighting a mismatch between caregiver readiness and clinical expectations. A recent systematic review by Determeijer et al. (2024) identified limited caregiver participation due to a lack of knowledge or difficulties managing complex care responsibilities [26]. Nurses in this study reported that caregivers often lacked the knowledge or confidence to engage effectively in fundamental roles, such as personal hygiene, household tasks, emotional support, transportation, and financial management.

One particularly concerning issue reported by nurses was adherence to infection control measures. While Fernandes et al. (2019) found that caregivers acknowledged the importance of hand hygiene, correct practices were inconsistently followed [27]. Similarly, Kaziunas et al. (2016) identified significant knowledge gaps among pediatric HSCT caregivers [18], underscoring the persistent need for targeted education and training.

The transitional role caregivers undergo – from passive family support to active care providers- was emphasized by participants. This transformation, as previously described by Kaziunas et al. (2016), includes learning to navigate the healthcare system, managing complex care at home, and developing communication strategies with clinical staff. However, caregivers often struggle to meet these evolving demands, contributing to a gap between nurses’ expectations and actual caregiver engagement [18].

This study highlights the positive effects of the caregiver engagement process, including reduced discharge time, improved patient hygiene, and enhanced psychological well-being. These findings are consistent with Levoy et al.’ study (2023), which demonstrated that incorporating caregiver involvement resulted in significantly lower rates of all-cause rehospitalizations within two months, compared to those lacking caregiver involvement [28]. Additionally, numerous studies [29, 30] have underscored the importance of caregiver engagement in providing psychological support, which significantly improves patient well-being and overall treatment outcomes. However, this study also reveals potential risks associated with insufficient/excessive caregiver involvement. As stated by Bevans & Sternberg (2012), and Elliot et al.’s study both lack of caregiver and over-involvement can lead increased stress for both patients and caregivers, and along with potential conflicts within the care team [31, 32]. Levoy et al.’s meta-analysis emphasizes the importance of balancing caregiver involvement to achieve optimal patient care and outcomes [28]. While caregiver engagement can enhance patient outcomes, excessive involvement can lead to caregiver burnout, whereas insufficient participation may result in unmet needs, poor patient outcomes, and increased hospital readmissions [18, 30].

Another significant finding of this study is the challenges and barriers experienced by caregivers in the HSCT process. Caregiver exhaustion, both physical and psychological, emerged as a primary concern. Nurses’ observations of signs of caregiver fatigue align with existing literature on caregiver strain, including studies [10, 15, 33], which report high levels of fatigue among caregivers, particularly in long-term caregiving settings. Additionally, the psychological burden of caregiving in high-intensity environments such as HSCT has been well-documented. As highlighted by Yang et al. (2025), informal caregivers experience significant psychosocial burden and have unmet needs before HSCT, emphasizing the necessity for targeted support interventions [34]. Social and cultural barriers also posed as major obstacles to caregiver engagement. Cultural differences, family conflicts, and language barriers were commonly cited difficulties, consistent with findings from studies [35–37]. The findings of this study underscore the multifaceted challenges faced by caregivers in the HSCT process. Caregiver exhaustion, both physical and psychological, is a significant concern, particularly in high-demand caregiving environments. Also, cultural misunderstandings, family conflicts, and language barriers not only affect the caregiver’s ability to provide optimal care but also strain the communication between caregivers, patients, and healthcare providers.

The study further emphasized the critical role of nurses in supporting caregivers. Nurses recognized the importance of emotional support in helping caregivers manage stress. Additionally, they noted the need for caregiver education on essential skills such as hygiene management, medication adherence, and technical aspects of care. Kazuinas et al. proposed five areas of support for caregiver involvement, similar to our study: analyzing laboratory results, following medications, identifying healthcare providers, providing information about clinical trials, and developing outpatient management skills [18]. Caregiver-defined needs and support mechanisms vary based on caregivers’ own interpretations rather than their assessed and stated needs [38]. Given the critical role of caregivers and the potential risk of their needs being unmet, implementing a person-centered, individualized, and standardized system for needs assessment and systematic support could be highly beneficial [39].

The role of nurses in supporting caregivers was also a key focus of this study, as they actively participated in providing both practical assistance and emotional support. The ability of nurses to establish positive relationships with caregivers and provide relief was particularly emphasized, with respite care emerging as a critical theme. In the study by Hagedoorn et al., nurse-provided education and guidance were found to reduce caregiver stress and improve the quality of care, particularly in assisting caregivers with medical tasks in HSCT [40]. Similarly, Fauer et al. highlighted that both patients and caregivers adapted more effectively to the treatment process when they received structured education and support services from nurses [41]. These findings emphasized the essential role of nurses not only in in-patient care but also in empowering and supporting caregivers throughout the treatment journey.

### Strengthens and limitations

This study has several strengths, including a qualitative descriptive design, which enabled the collection of rich, in-depth data, providing valuable insights into the subjective nature of nurses’ experiences and perspectives. The involvement of researchers with both clinical and academic expertise ensures that a strong understanding of both the clinical context of HSCT and qualitative research methodologies informs the study. The findings of this study have implications for improving nursing strategies and caregiver support in HSCT care, which may contribute to better patient outcomes and enhanced caregiver well-being.

However, this study also has several limitations. The inclusion of only eleven nurses from a single center may limit the generalizability of the findings. A larger and more diverse sample, incorporating nurses from various healthcare settings, could have provided a broader perspective on caregiver involvement in HSCT care. Additionally, the study focused solely on the views of nurses and did not include the perspectives of caregivers or patients themselves. Including these voices would offer a more comprehensive understanding of the caregiving experience in HSCT, which could lead to more holistic recommendations for improving care.

## Conclusion

HSCT is a treatment method that involves a comprehensive continuum of care, spanning from the initial decision-making process to discharge, including home care. In this challenging time, this study highlights that nurses perceive caregiver engagement as a critical factor in its positive impact on patient outcomes and the challenges faced by caregivers (physical, emotional, and social challenges that may hinder their ability to care). Furthermore, nurses in this study emphasized the critical role they play in supporting caregivers through emotional support, education, and respite services. Enhancing caregiver support through education, psychological support, and improved communication can help alleviate the burden of caregiving and improve the well-being of both caregiver and patient.

## Supplementary Information

Below is the link to the electronic supplementary material.

Supplementary file1 (DOCX 22 KB)

## Data Availability

No datasets were generated or analysed during the current study.
